# Management of patients with early stage lung cancer – why do some patients not receive treatment with curative intent?

**DOI:** 10.1186/s12885-020-6580-6

**Published:** 2020-02-10

**Authors:** Ross Lawrenson, Chunhuan Lao, Leonie Brown, Lucia Moosa, Lynne Chepulis, Rawiri Keenan, Jacquie Kidd, Karen Middleton, Paul Conaglen, Charles de Groot, Denise Aitken, Janice Wong

**Affiliations:** 1 Waikato Medical Research Centre, The University of Waikato, Level 3 Hockin building, Waikato Hospital, Hamilton, 3240 New Zealand; 2 Strategy and Funding, Waikato District Health Board, Hamilton, New Zealand; 3 Midland Cancer Network, Hamilton, New Zealand; 4 Taupua Waiora Research Centre, Auckland University of Technology, Auckland, New Zealand; 5 Respiratory Department, Waikato District Health Board, Hamilton, New Zealand; 6 Waikato Cardiothoracic Unit, Waikato District Health Board, Hamilton, New Zealand; 7 Radiation Oncology, Waikato District Health Board, Hamilton, New Zealand; 8 Respiratory Department, Lake District Health Board, Rotorua, New Zealand

**Keywords:** Lung cancer, Non-small cell lung cancer, Thoracic surgery, Stereotactic ablative body radiotherapy, Smoking

## Abstract

**Backgrounds:**

This study aims to understand the factors that influence whether patients receive potentially curative treatment for early stage lung cancer. A key question was whether indigenous Māori patients were less likely to receive treatment.

**Methods:**

Patients included those diagnosed with early stage lung cancer in 2011–2018 and resident in the New Zealand Midland Cancer Network region. Logistic regression model was used to estimate the odds ratios of having curative surgery/ treatment. The Kaplan Meier method was used to examine the all-cause survival and Cox proportional hazard model was used to estimate the hazard ratio of death.

**Results:**

In total 419/583 (71.9%) of patients with Stage I and II disease were treated with curative intent - 272 (46.7%) patients had curative surgery. Patients not receiving potentially curative treatment were older, were less likely to have non-small cell lung cancer (NSCLC), had poorer lung function and were more likely to have an ECOG performance status of 2+. Current smokers were less likely to be treated with surgery and more likely to receive treatment with radiotherapy and chemotherapy. Those who were treated with surgery had a 2-year survival of 87.8% (95% CI: 83.8–91.8%) and 5-year survival of 69.6% (95% CI: 63.2–76.0%). Stereotactic ablative body radiotherapy (SABR) has equivalent effect on survival compared to curative surgery (hazard ratio: 0.77, 95% CI: 0.37–1.61). After adjustment we could find no difference in treatment and survival between Māori and non-Māori.

**Conclusions:**

The majority of patients with stage I and II lung cancer are managed with potentially curative treatment – mainly surgery and increasingly with SABR. The outcomes of those being diagnosed with stage I and II disease and receiving treatment is positive with 70% surviving 5 years.

## Background

Lung cancer is the leading cause of cancer death in New Zealand [1]. Mortality in Māori, the indigenous people in New Zealand, is 2.6 times greater than in New Zealand Europeans [1]. Overall, outcomes from lung cancer in New Zealand are poor with a 5-year survival of only 11% [2]. This is mainly because the majority of lung cancer patients are diagnosed at late stage. In a recent study of lung cancer patients in our New Zealand region, only 16.5% were diagnosed with early stage (stage I and II) lung cancer [3].

Patients with early stage disease can be considered curable with successful surgery, or stereotactic ablative body radiotherapy (SABR) [4]. Some stage II and III patients also have successful outcomes with radical radiotherapy and chemo radiotherapy. Surgical resection rates for lung cancer vary between countries and even between centres in a particular country [5]. Overall, 14.7% of non-small cell lung cancer (NSCLC) patients receive surgery in New Zealand compared to 19.1% in Victoria, Australia [6, 7]. Previous studies in New Zealand have reported lower surgical rates in Māori [8]. New Zealand is looking to improve the proportion of lung cancer patients diagnosed with early stage through the use of an educational campaign. However, we have limited data on how early stage lung cancer is currently managed? There are also limited data on the outcomes of treatment of early stage disease.

The Midland Lung Cancer Register collects data from four District Health Boards (DHB) with a combined population of 800,000 residents. Tertiary lung cancer management is principally based at Waikato Hospital [3]. Waikato Hospital provides both surgical services and radiotherapy services for cancer patients for the region; with radiotherapy services also available in the Bay of Plenty DHB. This study aims to understand the factors that influence whether patients receive potentially curative treatment for their lung cancer, to understand which patients receive surgical management, and to examine the outcomes in those receiving surgery, other forms of treatment compared to those patients who receive palliative care.

## Methods

We analysed lung cancer data from the Midland Lung Cancer Register between January 2011 and December 2018 [3]. The Midland Lung Cancer Register is derived from data collected at multidisciplinary meetings (MDMs) within the region and complemented by data sourced from the New Zealand Cancer Registry (NZCR). Patients diagnosed with stage I and II lung cancer (ICD code: C33, C34) and resident in the Midland Cancer Network region (including Waikato, Lakes, Bay of Plenty and Tairawhiti District Health Board) in 2011–2018 were included. Patients that were not discussed at an MDM were identified by the NZCR, and missing data was included from examination of their clinical records. For those who did not have a record of treatment, patient notes were searched to ascertain the reasons for no treatment. These were categorised into: comorbidities, poor lung function, poor Eastern Cooperative Oncology Group (ECOG) performance status (2+) [9], high risk of surgical complications, patient refusal or unknown reasons.

Data collected on individual patients included age, sex, ethnicity, DHB of domicile, type of lung cancer (NSCLC, small cell, others and unknown), stage of cancer, lung function (FEV1 measurement), ECOG status, and presence of known co-morbidities as measured by Charlson Index [10]. We then identified the treatment received by patients, including curative surgery (lobectomy, partial resection of lung and pneumonectomy), curative radiotherapy (radical radiotherapy), SABR, curative chemo radiation or palliative treatment which could include palliative chemo radiation, palliative chemotherapy, or symptomatic palliative care only. Mortality data were derived from the Midland Lung Cancer Register, New Zealand Cancer Registry and hospital system (iPM) with a censor date of 25 June 2019. Statistical analyses were then performed on this Combined Lung Cancer Register.

In order to determine if there is an ethnic basis to inequity of care, patient demographics, tumour characteristics and treatment were compared between Māori and non-Māori patients. The difference was examined with Chi-square test. Reasons for not having potentially curative surgery as the primary treatment were also explored and classified into comorbidity, lung function problems, poor ECOG status, surgical complications, patient refusal and unknown/other reasons. Logistic regression model was used to estimate the odds ratios of having curative treatment for Māori patients compared with non-Māori patients after adjustment for patient demographics and tumour characteristics. We also examined the factors that influence whether patients received alternative curative treatment compared to surgery.

The Kaplan Meier method was used to examine the all-cause survival by treatment option and by ethnicity (Māori vs non-Māori). For survival analyses, patients without mortality information were considered to be censored on 25 June 2019. Cox proportional hazard model was used to estimate the hazard ratio of death for Māori compared to non-Māori after adjustment for age, sex, year of diagnosis, stage, comorbidities and treatments. All data analyses were performed in IBM SPSS statistics 25 (New York, United States).

## Results

The Combined Lung Cancer Register included 3331 resident cases (1050 Māori and 2281 non-Māori) between 2011 and 2018. This study included 583/3331 (17.5%) with early stage disease (Table 1). This was made up of 169/1050 (16.1%) Māori and 414/2281 (18.1%) non-Māori patients. Over 90% of the early stage patients were either a current smoker (30.9%) or ex-smoker (60.1%). Among the lung cancer patients, 47.7% had a history of chronic obstructive pulmonary disease (COPD). There were 452 cases of NSCLC, 14 cases of small cell lung cancer, and 106 patients did not have a pathology report. A record of unknown pathology was associated with significant comorbidities in 37 (34.9%) patients, frailty/high risk – ECOG 2+ 10 (9.4%) and very poor lung function 25 (23.6%). The 169 Māori patients were younger, more likely to be current smokers, have a diagnosis of COPD and have NSCLC-other and small cell lung cancer, and more likely to have FEV1 of < 50% than non-Māori patients.

**Table 1:** Patient demographics and tumour characteristics

Subgroup	Māori	Non-Māori	*P*-value (Chi-square test)	Total
Sex				
Female	104 (61.5%)	220 (53.1%)	0.064	324 (55.6%)
Male	65 (38.5%)	194 (46.9%)		259 (44.4%)
Age (years)				
< 60	35 (20.7%)	53 (12.8%)	< 0.001	88 (15.1%)
60–69	65 (38.5%)	120 (29.0%)		185 (31.7%)
70–79	57 (33.7%)	170 (41.1%)		227 (38.9%)
80+	12 (7.1%)	71 (17.1%)		83 (14.2%)
Smoking status				
Current smoker	63 (40.4%)	104 (27.0%)	< 0.001	167 (30.9%)
Ex-smoker	91 (58.3%)	234 (60.8%)		325 (60.1%)
Never smoked	2 (1.3%)	47 (12.2%)		49 (9.1%)
Unknown	13	29		42
Charlson Comorbidity				
0	37 (21.9%)	119 (28.7%)	< 0.001	156 (26.8%)
1	44 (26.0%)	147 (35.5%)		191 (32.8%)
2	51 (30.2%)	99 (23.9%)		150 (25.7%)
3	29 (17.2%)	33 (8.0%)		62 (10.6%)
4+	8 (4.7%)	16 (3.9%)		24 (4.1%)
COPD				
No	57 (39.9%)	207 (57.2%)	< 0.001	264 (52.3%)
Yes	86 (60.1%)	155 (42.8%)		241 (47.7%)
Unknown	26	52		78
Cell type				
NSCLC	124 (91.2%)	328 (96.2%)	< 0.001	452 (94.8%)
Others	2 (1.5%)	9 (2.6%)		11 (2.3%)
Small cell	10 (7.4%)	4 (1.2%)		14 (2.9%)
Unknown	33	73		106
FEV1				
< 50%	46 (30.3%)	63 (17.4%)	< 0.001	109 (21.2%)
50%~ 80%	70 (46.1%)	139 (38.4%)		209 (40.7%)
80%+	36 (23.7%)	160 (44.2%)		196 (38.1%)
Unknown	17	52		69
ECOG				
0	51 (32.3%)	157 (40.6%)	0.194	208 (38.2%)
1	73 (46.2%)	158 (40.8%)		231 (42.4%)
2+	34 (21.5%)	72 (18.6%)		106 (19.4%)
Unknown	11	27		38
Total	169	414		583

In total 419/583 (71.9%) of patients with Stage I and II disease were treated with curative intent - 272 (46.7%) patients had curative surgery, including 199 lobectomies, 59 partial resection of lung, and 14 pneumonectomy (Table 2). Another 64 (11.0%) patients were treated with SABR, 67 (11.5%) received curative radical radiotherapy, and 16 (2.7%) had curative chemo/radiotherapy. Amongst those not having curative treatment (164 (28.1%)), 33 (5.7%) had palliative radiotherapy, 14 (2.4%) had palliative chemotherapy, and 117 (20.1%) had best supportive care alone. Māori patients appeared to be less likely to have curative surgery (39.6% vs 49.5%, *p*-value = 0.027), but were as likely to have curative treatment as non-Māori patients (70.4% vs 72.5%, *p*-value = 0.618). The principal reason recorded in the patient records indicating why these lung cancer patients did not have curative treatment included significant comorbidities in 37 (22.6%) patients, 24 (14.6%) poor lung function, 24 (14.6%) poor ECOG status, 19 (11.6%) high risk of surgical complications, 16 (9.8%) patient refusal and 43 (26.2%) unrecorded.

**Table 2:** Primary treatment for lung cancer patients by ethnicity

Primary treatment	Māori	Non-Māori	Total
Curative surgery	67 (39.6%)	205 (49.5%)	272 (46.7%)
Lobectomy	49 (29.0%)	150 (36.2%)	199 (34.1%)
Partial resection of lung	16 (9.5%)	43 (10.4%)	59 (10.1%)
Pneumonectomy	2 (1.2%)	12 (2.9%)	14 (2.4%)
SABR	22 (13.0%)	42 (10.1%)	64 (11.0%)
Radical radiotherapy	23 (13.6%)	44 (10.6%)	67 (11.5%)
Curative chemo/radiotherapy	7 (4.1%)	9 (2.2%)	16 (2.7%)
Palliative radiotherapy	14 (8.3%)	19 (4.6%)	33 (5.7%)
Palliative chemotherapy	4 (2.4%)	10 (2.4%)	14 (2.4%)
Supportive care	32 (18.9%)	85 (20.5%)	117 (20.1%)
Total	169	414	583

The logistic regression model showed that age, year of diagnosis, cancer stage, cancer cell type, FEV1 and ECOG status had an impact on the likelihood of having curative treatment (Table 3). Patients who were younger, were diagnosed in more recent years, had stage I disease, had NSCLC, had FEV1 of 80%+, and had an ECOG score of 0 were more likely to receive curative treatment. Amongst those who received curative treatments, younger patients were more likely to have surgery as the primary treatment (odds ratio: 0.91, 95%:0.87–0.95). Current smokers and ex-smokers were less likely to have surgery and more likely to be treated with radiotherapy and chemotherapy than people who never smoked (respective odds ratio: 0.11 (95% Confidence interval (CI): 0.02–0.46); 0.23 (95% CI: 0.06–0.89)). Patients who had NSCLC, had FEV1 of 80%+, and had an ECOG score of 0 were more likely to undergo surgery. After adjustment for other factors we did not find a difference in access to curative treatment and curative surgery between Māori and non-Māori patients (respective odds ratio: 0.80 (95% CI: 0.46–1.38); 1.03 (95% CI: 0.53–2.00)).

**Table 3:** Adjusted odds ratios from logistic regression model

Subgroup	Having curative treatment vs no curative treatment			Having surgery vs other curative treatment		
	Odds ratio	95% CI	*p*-value	Odds ratio	95% CI	*p*-value
Sex						
Female	Ref			Ref		
Male	0.85	(0.51 - 1.41)	0.536	1.26	(0.70 - 2.29)	0.446
Ethnicity						
Māori	0.80	(0.46 - 1.38)	0.424	1.03	(0.53 - 2.00)	0.932
Non-Māori	Ref			Ref		
Age (Continuous)	0.92	(0.89 - 0.95)	< 0.001	0.91	(0.87 - 0.95)	< 0.001
Smoking status						
Current smoker	0.40	(0.12 - 1.38)	0.149	0.11	(0.02 - 0.46)	0.003
Ex-smoker	0.49	(0.15 - 1.57)	0.229	0.23	(0.06 - 0.89)	0.033
Never smoked	Ref			Ref		
Charlson Comorbidity						
0	Ref			Ref		
1	0.82	(0.36 - 1.87)	0.644	0.43	(0.17 - 1.11)	0.080
2	1.06	(0.45 - 2.50)	0.902	0.35	(0.13 - 0.93)	0.035
3	0.93	(0.34 - 2.54)	0.894	0.20	(0.06 - 0.73)	0.015
4+	0.55	(0.15 - 2.02)	0.365	0.10	(0.02 - 0.56)	0.009
Year of diagnosis (Continuous)	1.15	(1.03 - 1.28)	0.015	0.75	(0.65 - 0.86)	< 0.001
Stage						
I	Ref			Ref		
II	0.29	(0.17 - 0.48)	< 0.001	0.58	(0.31 - 1.08)	0.088
Cell type						
NSCLC	Ref			Ref		
Others	0.29	(0.17 - 0.51)	< 0.001	0.08	(0.03 - 0.23)	< 0.001
FEV1						
< 50%	0.35	(0.16 - 0.77)	0.009	0.04	(0.01 - 0.12)	< 0.001
50%~ 80%	0.70	(0.36 -\ 1.38)	0.304	0.42	(0.20 - 0.85)	0.016
80%+	Ref			Ref		
ECOG						
0	Ref			Ref		
1	0.52	(0.26 - 1.02)	0.056	0.57	(0.30 - 1.09)	0.091
2+	0.13	(0.06 - 0.26)	< 0.001	0.14	(0.05 - 0.40)	< 0.001

**Fig. 1:**
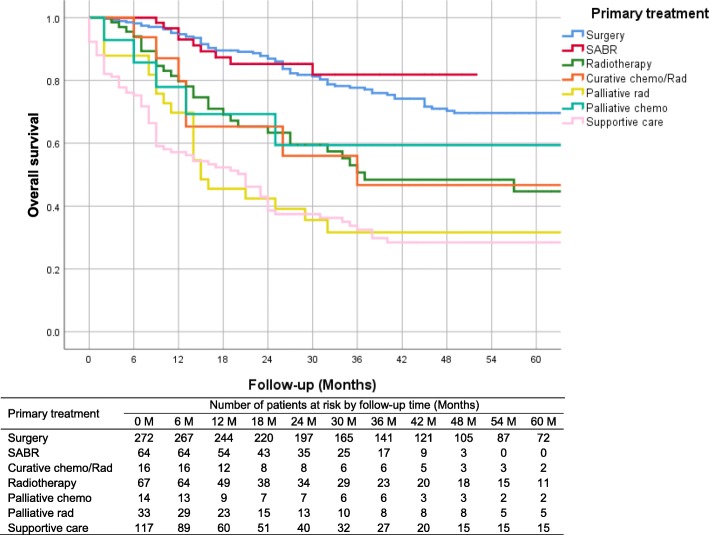
All-cause survival by treatment option

There were 217 deaths (37.3%) in this cohort with a median follow-up time of 27 months and a mean follow-up time 34 months. Outcomes in patients with stage I and II lung cancer varied depending on the treatment received (Fig. 1). Those who were treated with surgery had a 2-year survival of 87.8% (95% CI: 83.8–91.8%) and 5-year survival of 69.6% (95% CI: 63.2–76.0%). SABR has only been available in the region since mid 2015 but outcomes are similar to surgery in the first 2 years post treatment (2-year survival: 85.2, 95% CI: 75.8–94.7%, log-rank test *p*-value = 0.556). Prior to the use of SABR, some patients were offered radiotherapy with curative intent and in this group of patients 2-year survival is only 65.3% (95% CI: 53.1–77.4%) and 5-year survival was 50%. Patients offered palliative treatment only had a 2-year survival of 45.0% (95% CI: 37.0–53.0%) and 5-year survival of 31.8% (95% CI: 23.9–39.6%).

**Fig. 2:**
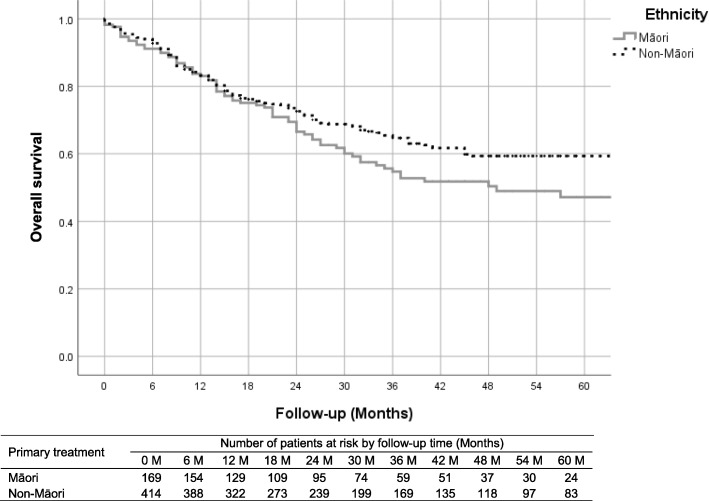
All-cause survival between Māori and non-Māori

Māori patients had a similar survival to non-Māori patients (Fig. 2, Log-rank test p-value = 0.091). The 2-year and 5-year survival for Māori patients was 69.4% (95% CI: 62.2–76.7%) and 47.1% (95% CI: 37.8–56.4%), compared to 73.5% (95% CI: 69.1–77.9%) and 59.3% (95% CI: 53.9–64.8%) for non-Māori patients.

The hazard ratio (Table 4) of all-cause mortality for Māori patients compared to non-Māori patients was 1.25 (95% CI: 0.92–1.69, p-value = 0.150). SABR has equivalent effect on survival compared to curative surgery (hazard ratio: 0.77, 95% CI: 0.37–1.61). The all-cause survival for stage I and II lung cancer patients has improved over time (hazard ratio: 0.94, 95% CI: 0.87–1.00).

**Table 4:** Adjusted hazard ratio for overall survival from Cox proportional hazard model

Factors	Hazard ratio	95% CI of hazard ratio	*P*-value
Sex			
Female	Ref		
Male	1.21	(0.91 - 1.62)	0.195
Ethnicity			
Māori	1.25	(0.92 - 1.69)	0.151
Non-Māori	Ref		
Age (Continuous)	1.01	(0.99 - 1.03)	0.172
Charlson Comorbidity			
0	Ref		
1	1.10	(0.70 - 1.75)	0.673
2	1.07	(0.66 - 1.73)	0.794
3	1.02	(0.55 - 1.86)	0.959
4+	1.19	(0.58 - 2.44)	0.640
Smoking status			
Current smoker	2.51	(1.06 - 5.94)	0.037
Ex-smoker	2.24	(0.96 - 5.22)	0.061
Never smoked	Ref		
Year of diagnosis (Continuous)	0.94	(0.87 - 1.00)	0.068
Stage			
I	Ref		
II	1.35	(1.02 - 1.80)	0.039
Primary treatment			
Curative surgery	Ref		
SABR	0.77	(0.37 - 1.61)	0.486
Radiotherapy	1.94	(1.23 - 3.07)	0.005
Curative chemo/Rad	1.80	(0.80 - 4.03)	0.153
Palliative rad	2.89	(1.72 - 4.85)	< 0.001
Palliative chemo	1.69	(0.66 - 4.32)	0.270
Supportive care	3.35	(2.26 - 4.96)	< 0.001

## Discussion

We found that 71.9% of early stage lung cancer patients in our region were treated with potentially curative treatment. The commonest form of treatment was surgery. Thus in the Midlands Region a total of 272/3331 (8.2%) of lung cancer patients were treated with curative surgery. This low rate of surgical treatment is similar to that found in the UK but lower than the rate reported in Australia and some European countries [11, 12]. While the advent of SABR has coincided with an increasing proportion of early stage patients being offered curative treatment, significant improvement will only be achieved when the proportion of patients with early stage disease at diagnosis is increased. This can either be achieved through greater awareness of symptoms of lung cancer e.g. through social media campaign [13] and through the introduction of lung cancer screening [14, 15].

We have shown that there are a number of reasons why patients do not receive curative treatment. Overall, less than half of patients with stage I and II disease in our region 272/583 (46.7%) were treated with surgery. This figure has not improved from the findings in a similar New Zealand study in 2004 which reported a surgery rate of 56% of stage I and II NSCLC [7]. Another 147/583 (25.2%) of patients in our study were treated with alternative potentially curative treatment while 164/583 (28.1%) were treated with palliative care only. Patients with stage I and II NSCLC receiving palliative care were older than those who had curative treatment (mean age of 73 years vs 68 years). Other reasons included cancer stage –(stage II cases were less likely to be treated curatively than stage I), cancer cell type (small cell tumours were less likely to be treated than NSCLC), and those with COPD or poor respiratory function who were less likely to receive surgery or curative treatment as were those with a poor ECOG status. These findings are similar to the findings from a Danish study [16] of stage I lung cancer and the historical New Zealand study [7].

SCLC proliferates more rapidly and has a high propensity to metastasise. Most cases will present with locally advanced or metastatic disease. On rare occasions, patients are identified with small cell lung cancer (SCLC) histology but with early stage disease potentially suitable for resection [17]. Overall our cohort had 440/3331 (13%) small cell lung cancers. There were only 14 small cell lung cancer cases in our group of stage I and II diseases, and only one had curative surgery. In a large cohort of 45,848 patients with SCLC only 1% were treated surgically [18]. The 5-year survival in this cohort from the turn of the century was only 31% and the HR compared with NSCLC was 1.47 [18]. Our findings suggest that surgical intervention for SCLC is a rare event, partly because few cases present with early stage disease and other treatment modalities are more likely to be taken up.

Our study also shows that patients who identify as Māori are less likely to receive curative surgical resection of stage I and II lung cancer than those who do not identify as Māori. This finding was based on the unadjusted analysis, and the difference disappeared after adjustment for other factors. This could suggest that the New Zealand healthcare system is ensuring equity of access to curative surgical resection for patients. Māori presenting with early stage disease are younger than non-Māori, and more likely to have COPD, be a current smoker, have an FEV1 less than 50% and have small cell histology. Māori generally have lower socioeconomic status which is associated with poor surviva [19, 20]. After adjustment for these factors it appears that Māori are not less likely to receive curative treatment (odds ratio 0.80, 95% CI 0.46–1.38) or surgery (odds ratio 1.03, 95% CI: 0.53–2.00). It maybe this finding is a Type 2 error and if we had a bigger sample then potentially we might show a difference. This means that we need to continue to monitor access to curative treatments for Māori if we are to reduce the inequities in outcomes that we know are present [7, 21].

Our findings show that the all-cause survival from surgery in this group of patients are 85% at 2 years and 70% at 5 years. This is similar to the survival reported in a 2004 study where the 2-year survival was 81% [7]. This supports the assertion that early stage lung cancer can be “cured” [22]. Indeed if we look just at the 199 NSCLC patients treated with lobectomy we find the 5-year survival is over 70% which is comparable with the findings from studies in major centres in the USA [22].

Patients with stage I and II NSCLC treated with SABR have comparable outcomes to those treated with surgery. This is despite the finding that those treated with curative intent with SABR tended to have additional risk factors including older age, higher ECOG status score, more comorbidities and more smokers. The numbers of patients treated with SABR is relatively small and further follow up of a greater number of patients is needed to confirm this finding but the initial results are very encouraging. A systematic review [23] reported that the overall and cancer-specific survival between SABR and lobectomy for stage I NSCLC were similar after 1-year follow-up, but lobectomy appeared to have more favourable outcomes after 3-year and 5-year follow-up. However, this systematic review only included one randomised clinical trial [24] and the other six included studies were cohort studies which may have been subject to bias [25–30].

Outcomes in patients managed with palliative care are relatively poor where only 30% of palliative patients with Stage I and II disease are surviving 5 years. Many of these patients are older and have significant comorbidities which impact on overall survival. The small number of patients offered palliative chemotherapy appear to be doing better with a 2-year survival of 60%. However, overall survival in this group of patients managed with supportive/palliative care is higher than previous reports. Stevens et al. [7] only showed 20% survival with 2 years follow-up, while our study has shown similar improvement year by year during the study period.

One of the strengths of our study is that it was based on the comprehensive lung cancer register, with relatively complete data on patient demographics, tumour characteristics and treatment. The limitations of this study included the small number of patients and short follow-up time in some treatment group, e.g. SABR. Also as an observation study, this study is prone to selection bias.

## Conclusions

The majority of patients with stage I and II lung cancer are managed with potential curative treatment – mainly surgery and increasingly SABR. After adjustment for key variables such as smoking, comorbidities and lung function status, the likelihood of Māori patients having curative treatment was similar to non-Māori. This suggest that outcomes for Māori patients can be improved by addressing smoking and the management of comorbidities. While the outcomes of those being diagnosed with stage I and II disease and receiving treatment is positive with 70% surviving 5 years, the next target is to substantially increase the population of lung cancer patients diagnosed with early stage disease.

## Data Availability

The datasets generated and/or analysed during the current study are not publicly available due the confidentiality of patient data but are available from the corresponding author on reasonable request.

## Abbreviations

CI: Confidence interval
COPD: Chronic obstructive pulmonary disease
DHB: District Health Boards
ECOG: Eastern Cooperative Oncology Group
FEV1: Forced Expiratory Volume
MDM: Multidisciplinary meeting
NSCLC: Non-small cell lung cancer
NZCR: New Zealand Cancer Registry
SABR: Stereotactic ablative body radiotherapy
SCLC: Small cell lung cancer
